# The effects of parental respect for children’s decision-making and respect for human rights on depression in early adolescents: The mediating effect of self-esteem

**DOI:** 10.1371/journal.pone.0300320

**Published:** 2024-04-04

**Authors:** Sangmi Lee

**Affiliations:** College of Nursing, Dongyang University, Yeongju-si, Gyeongsangbuk-do, Republic of Korea; Birla Institute of Technology and Science, INDIA

## Abstract

This study investigated the structural relationships among parental respect for children’s decision-making, respect for human rights, and self-esteem, and their impact on depression in early adolescents. The study utilized data from 2,747 middle school students who participated in the 2020 Survey on the Current Status of Korean Children’s and Youth’s Rights conducted by the National Youth Policy Institute. The data were analyzed using a structural equation model based on partial least squares with SmartPLS 3.0. The analysis revealed that both parental respect for children’s decision-making and respect for human rights perceived by middle school students had a significant positive impact on self-esteem and a significant negative impact on depression. Furthermore, self-esteem was found to have a significant negative effect on depression. Importantly, self-esteem also played a significant mediating role in the relationship between parental respect for children’s decision-making and depression, as well as the relationship between respect for human rights and depression. Therefore, in order to manage depression, it is necessary to develop strategies that encourage parental respect for children’s decision-making, promote respect for human rights, and foster self-esteem in early adolescents.

## Introduction

Depression is an emotional disorder can emerge during adolescence, and it is a significant contributor to both physical and mental illness and disability, potentially impacting adulthood [1]. Recent data indicate an increasing trend in the number of Korean adolescents experiencing depression [2]. In fact, studies have found that 27% of Korean adolescents report high levels of depression, characterized by feelings of sadness or depression without a discernible cause [3]. Depression is a potent risk factor that diminishes life satisfaction [4, 5] and increases the likelihood of suicidal thoughts [6] in adolescents. Early adolescence is a time when individuals often experience emotional turmoil along with rapid physical changes related to secondary sexual characteristics [7]. Therefore, this is a time of particular concern as an increase in depression has been observed, but there are still many ambiguities about prevention and solutions for depression in early adolescence [1, 8, 9]. Consequently, a variety of approaches are necessary to understand and address depression in early adolescence.

Environmental factors have a significant impact on depression in early adolescents [8], and parenting attitudes are a particularly crucial element. Prior research has demonstrated that parenting behaviors exert a more substantial influence on adolescent depression than negative peer relationships or family and regional characteristics [10, 11]. Furthermore, abusive or neglectful parenting attitudes have been identified as highly significant risk factors for depression [10, 11]. Specifically, a parenting attitude that respects children’s decision-making—defined as including children in the decision-making process and valuing their opinions—has a considerable impact on the emotional well-being of adolescent children [12]. Previous studies have indicated that adolescents who perceive their parents as receptive to their opinions and respectful of their autonomy are less likely to experience depression [13]. This suggests a correlation between a parenting attitude that respects children’s decision-making and depression levels among adolescents.

Human rights are fundamental and inherent to all individuals. The Universal Declaration of Human Rights explicitly asserts that every human being is entitled to their dignity and rights, irrespective of race, nationality, gender, religion, class, or status [14]. However, children and adolescents are particularly susceptible to human rights violations due to their physical and psychological dependence on adults, their developmental characteristics, and their economic vulnerability [15]. In response to this, the United Nations Convention on the Rights of the Child was adopted in 1989 to safeguard the dignity and rights of children. Korea ratified this convention in 1991 [15]. As a result, Korea is actively monitoring the implementation of the UN Convention on the Rights of the Child. Each year, the Korea Youth Policy Institute conducts a national survey on the human rights situation of children and adolescents and reports the findings [3]. According to the most recent survey (2020) on the human rights of children and adolescents, there is an increasing trend in respect for human rights, as evaluated in terms of perceptions of how much one’s human rights are respected in social settings such as home and school [3].

The level of respect for human rights is associated with depression in young people. Previous studies have found that young people who perceive a high level of respect for human rights in their homes and schools are less likely to experience depression [5, 16, 17]. Conversely, instances of human rights violations, such as parental abuse or neglect [16, 18, 19], as well as experiences of school violence or cyber victimization [17, 20], have been linked to an increase in depression. Furthermore, racial discrimination has been identified as a significant predictor of depressive symptoms in adolescents [21, 22].

Self-esteem, defined as a positive feeling or evaluation of one’s own worth [23], has been identified as a significant factor in mitigating depression [24, 25]. Previous research has demonstrated that high self-esteem during adolescence can decrease the likelihood of depression [13], whereas low self-esteem can contribute to its onset [13, 26, 27]. Notably, self-esteem during adolescence has been reported as a significant predictor of depression in adulthood [28, 29], indicating that adolescent self-esteem plays an important role in influencing depression after adolescence.

As cognitive abilities develop during adolescence, the ability to objectively evaluate oneself gradually improves, which often results in a decrease in self-esteem [30]. Parenting attitudes, such as acceptance, play a significant role in the development of self-esteem during this period [13, 31]. Specifically, when parents respect their adolescent children’s decision-making, it allows the children to perceive that their worth is acknowledged [32, 33], thereby enhancing their self-esteem [33]. Moreover, numerous studies have consistently found that adolescents who feel their human rights are respected have higher self-esteem [33–35]. Conversely, experiences of cyber human rights violations have been associated with lower self-esteem among young people [36, 37]. Consequently, it is hypothesized that self-esteem could mediate the impact of parental respect for children’s decision-making and respect for human rights on depression.

A comprehensive review of the relevant research demonstrates that few previous studies have addressed the effects of parental respect for children’s decision-making and respect for human rights on early adolescents’ depression. In particular, no studies have investigated the mediating role of self-esteem in the correlation between young people’s experiences of parental respect for children’s decision-making or human rights and depression. Moreover, adequately explaining depression in early adolescents necessitates exploring the structural relationships among all four variables in combination. However, these associations have not been thoroughly investigated. An exploration of these structural relationships would help elucidate the complex and dynamic interplay between adolescent depression and factors related to human rights.

**Fig 1:**
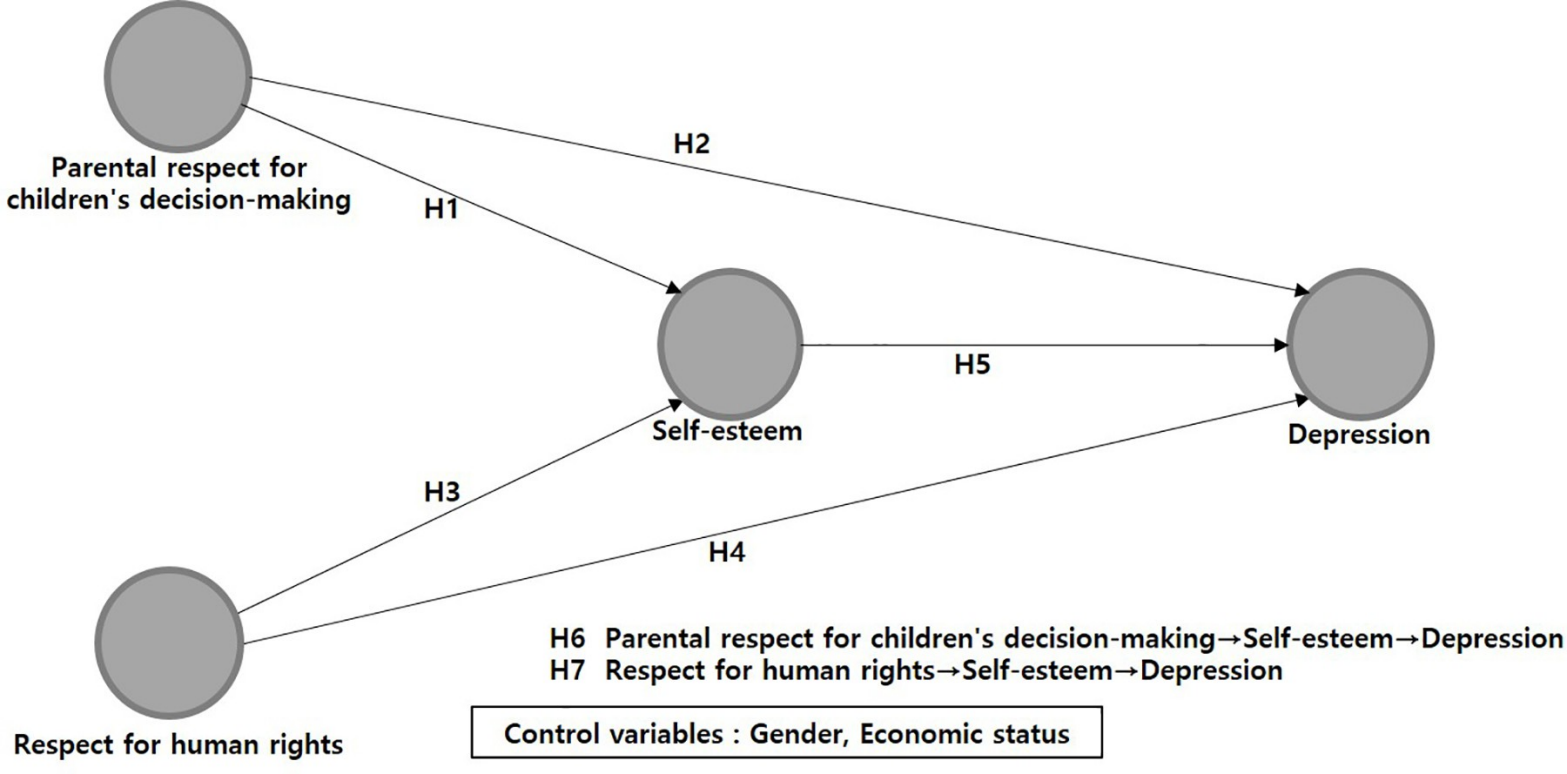
Research model.

Meanwhile, numerous prior studies have indicated significant differences in adolescent self-esteem and depression based on gender. Specifically, female students are at a higher risk of experiencing low self-esteem compared to their male counterparts [12, 30, 32]. Correspondingly, they also exhibit a higher risk of depression [16, 17]. In addition, previous research has demonstrated significant differences in adolescent self-esteem and depression based on their family’s economic status. Specifically, a family’s economic status has been found to have a significant positive correlation with adolescent self-esteem [12]. Moreover, a lower perceived economic status is associated with a significantly higher average depression score among adolescents [17]. Given these findings, this study investigated the pathway from parental respect for children’s decision-making and respect for human rights to depression, mediated through self-esteem in early adolescents, with gender and economic status serving as control variables. The following hypotheses were developed based on the findings of previous studies, and the research model (Fig 1) illustrates the proposed relationships between the variables in this work.

**Hypothesis 1 (H1).** Parental respect for children’s decision-making will have a positive effect on self-esteem in early adolescents.
**Hypothesis 2 (H2).** Parental respect for children’s decision-making will have a negative effect on depression in early adolescents.
**Hypothesis 3 (H3).** Respect for human rights perceived by early adolescents will have a positive effect on their self-esteem.
**Hypothesis 4 (H4).** Respect for human rights perceived by early adolescents will have a negative effect on their depression.
**Hypothesis 5 (H5).** Self-esteem will have a negative effect on depression in early adolescence.
**Hypothesis 6 (H6).** Early adolescents’ self-esteem will have a negative mediating effect on the relationship between parental respect for children’s decision-making and depression.
**Hypothesis 7 (H7).** Early adolescents’ self-esteem will have a negative mediating effect on the relationship between respect for human rights and depression.

## Methods

### Study design

This study involved a secondary data analysis, utilizing data from the 2020 Children and Adolescent Human Rights Survey conducted by the National Youth Policy Institute (NYPI) to explore the structural relationship between early adolescent depression, parental respect for children’s decision-making, respect for human rights, and self-esteem.

### Subjects and data collection

In this study, the target population comprised early adolescents, with data drawn from 2,747 middle school student participants in the 2020 Survey on the Current Status of Korean Children’s and Youth’s Rights, conducted by the National Youth Policy Institute. The 2020 Survey on the Current Status of Korean Children’s and Youth’s Rights is an annually repeated cross-sectional survey, initiated in 2009 by the Korea Youth Policy Institute. The survey’s purpose is to facilitate the implementation of the United Nations Convention on the Rights of the Child and to investigate the status and degree of change in the human rights of children and adolescents in Korea. The 2020 data utilized in this study were collected between July and October 2020 [3].

The 2020 Survey on the Current Status of Korean Children’s and Youth’s Rights targeted students from the 4th grade of elementary school to the 3rd grade of high school nationwide. The sample was drawn using stratified multi-stage cluster sampling, with the 2019 Statistical Yearbook of Education, published by the Ministry of Education, serving as the sampling frame. The survey was completed by targeting 8,623 individuals through a combination of group interviews and mail surveys [3]. The 2020 survey data utilized in this study is the most recent open data, accessible via the Korea Children and Youth Data Archive website (https://www.nypi.re.kr/archive/mps). For this study, after agreeing to apply for data through the aforementioned website, and providing details such as researcher type, major, and data use plan, the data (including the codebook, questionnaire, and data) were downloaded on July 22, 2023, and used to August 31, 2023.

### Ethical considerations

This study was carried out using publicly accessible, pre-existing data that had been processed to ensure the anonymity of the research participants. Approval for review exemption was obtained from the Institutional Review Board (IRB No.:1041495-202308-HR-02-01) prior to the commencement of the study. The data were sourced from the 2020 Survey on the Current Status of Korean Children’s and Youth’s Rights conducted by the NYPI in Korea (https://www.nypi.re.kr/archive/mps).

### Measurements

All measurements for this study used the scale developed by the NYPI [3]. The dependent variable was depression, the independent variables were parental respect for children’s decision-making and respect for human rights, and the mediator was self-esteem. Additionally, the control variables in this study were gender and economic level.

#### Depression

Depression was evaluated using questions pertaining to experiences of depression over the previous year. The scale comprises three questions: “I have felt unreasonably lonely,” “I have felt unreasonably anxious,” and “I have felt unreasonably sad or depressed.” Each item was rated on a 4-point Likert scale, with 1 representing “not at all” and 4 representing “very much.” A higher score signifies a greater degree of depression. In this study, the tool’s Cronbach’s α was .890.

#### Parental respect for children’s decision-making

Parental respect for children’s decision-making was assessed using questions about parents’ respectful approach for their children’s needs or opinion when making decisions on behalf of their children. This scale comprises four questions: “My parents (guardians) listen to and respect my opinions when deciding on important family issues,” “’When deciding which higher school to attend (high school, university, etc.), my parents (guardians) respect my school preference,” “When deciding on my future aspirations, my parents (guardians) respect my desired career path (occupation),” and “My parents (guardians) listen to and respect my opinion when deciding on study time and methods (choosing academies or study materials, etc.).” Each item was measured on a 4-point Likert scale, ranging from 1 (“not at all”) to 4 (“very much”). A higher score indicates a greater level of parental respect for children’s decision-making. In this study, the Cronbach’s α of the tool was .822.

#### Respect for human rights

The level of respect for human rights was evaluated using a series of questions aimed at understanding the perceptions of youth regarding their human rights in various environments, such as home, school, society, and cyberspace. This scale contains four questions, each asking “To what extent do you feel your human rights are respected?” in the following contexts: at home, at school, within the broader society, and in digital spaces like the internet. Responses were measured using a 4-point Likert scale, with 1 representing “not respected at all” and 4 indicating “very respected.” A higher score on this scale signifies a greater degree of perceived respect for human rights. The Cronbach’s α reliability coefficient for this tool, as used in this study, was .755.

#### Self-esteem

Self-esteem was evaluated using a series of questions that focused on an individual’s perception of their own value and qualities over the past year. This scale comprises three self-reflective questions: “I believe I am a valuable person,” “I believe I possess many good qualities (strengths),” and “I don’t have much to be proud of.” The final question was reverse-coded for this study. Each question was rated on a 4-point Likert scale, with 1 representing “not at all” and 4 representing “very much.” A higher score indicates a higher level of self-esteem. The reliability of this tool, as measured by Cronbach’s α, was .793 in this study.

#### Control variables

In this study, the control variables were gender and perceived economic status. Gender was categorized as a binary variable, with male represented by 0 and female by 1. Perceived economic status was coded as “high = 1,” “middle = 2,” and “low = 3,” reflecting adolescents’ perception of their family’s economic level. However, for the purposes of this study, these codes were reversed to “high = 3,” “middle = 2,” and “low = 1.” A higher score thus indicates a higher perceived economic status.

### Data analysis

In this study, data analysis was performed using SPSS 28.0 (IBM Corp., Armonk, NY, USA) and SmartPLS 3.0 (SmartPLS GmbH, Germany). The general characteristics of the study subjects were calculated as frequency and percentage, and the characteristics of the main variables were calculated as the mean and standard deviation. Skewness and kurtosis were also calculated for these variables, and the correlation between main variables was calculated using Pearson correlation coefficients. The structural relationship of the main variables in this study was analyzed using structural equation modeling (SEM) based on partial least squares (PLS), and the mediating effect of self-esteem was analyzed using the bootstrapping method.

## Results

### General characteristics of the participants

Of the 2,747 participants in this study, 1,425 (51.9%) were male. The majority were third-year middle school students, accounting for 951 (34.6%) of the total, although the distribution across the first to third years in middle school was relatively even. When asked about their perceived economic status, the majority, 1,552 participants (56.5%), reported “high,” while the fewest, 173 participants (6.3%), responded “low” (Table 1).

**Table 1:** General characteristics of participants (N = 773).

Characteristics		n (%)
**Gender**	**Male**	1,425 (51.9)
	**Female**	1,322 (48.1)
**Year in middle school**	**First**	915 (33.3)
	**Second**	881 (32.1)
	**Third**	951 (34.6)
**Subjective economic status** *	**High**	1,552 (56.5)
	**Medium**	1,004 (36.5)
	**Low**	173 (6.3)

*Including missing values

### Descriptive statistics and correlational analysis of the major variables

The average scores for the major variables were as follows: parental respect for children’s decision-making ranged from 3.32 points (±0.71) to 3.48 points (±0.64), respect for human rights ranged from 3.05 points (±0.69) to 3.51 points (±0.59), self-esteem ranged from 2.89 points (±0.84) to 3.08 points (±0.81), and depression ranged from 1.84 points (±0.95) to 1.91 points (±0.94). There was a statistically significant positive correlation between all relationships involving parental respect for children’s decision-making, respect for human rights, and self-esteem. Conversely, depression showed a statistically significant negative correlation with parental respect for children’s decision-making, respect for human rights, and self-esteem, as presented in Table 2.

**Table 2:** Descriptive statistics and correlation analysis of major variables.

Correlation		Parental respect for children’s decision-making				Respect for human rights				Self-esteem			Depression		
		➀	➁	➂	➃	➀	➁	➂	➃	➀	➁	➂	➀	➁	➂
**Parental respect for children’s decision-making**	**➀**														
	**➁**	.54**													
	**➂**	.51**	.63**												
	**➃**	.51**	.52**	.51**											
**Respect for human rights**	**➀**	.51**	.41**	.42**	.41**										
	**➁**	.30**	.27**	.27**	.27**	.52**									
	**➂**	.24**	.19**	.19**	.22**	.34**	.54**								
	**➃**	.15**	.13**	.14**	.13**	.26**	.39**	.52**							
**Self-esteem**	**➀**	.25**	.22**	.24**	.19**	.33**	.26**	.20**	.13**						
	**➁**	.25**	.21**	.23**	.21**	.31**	.26**	.22**	.12**	.74**					
	**➂**	.26**	.25**	.23**	.22**	.31**	.26**	.26**	.18**	.40**	.54**				
**Depression**	**➀**	-.17**	-.14**	-.14**	-.14**	-.26**	-.25**	-.27**	-.17**	-.17**	-.22**	-.40**			
	**➁**	-.19**	-.17**	-.17**	-.14**	-.25**	-.24**	-.27**	-.18**	-.20**	-.22**	-.39**	.70**		
	**➂**	-.15**	-.14**	-.13**	-.12**	-.24**	-.23**	-.25**	-.16**	-.20**	-.23**	-.40**	.75**	.74**	
**Mean**		3.33	3.45	3.48	3.32	3.51	3.34	3.07	3.05	3.08	2.89	2.97	1.91	1.84	1.87
**SD**		0.68	0.63	0.64	0.71	0.59	0.56	0.65	0.69	0.81	0.84	0.86	0.94	0.95	0.97
**Skewness**		-0.74	-0.89	-1.03	-0.82	-0.90	-0.28	-0.52	-0.64	-0.73	-0.42	-0.35	0.56	0.70	0.65
**Kurtosis**		0.35	0.57	0.87	0.32	0.74	0.26	0.91	0.92	0.21	-0.39	-0.75	-0.89	-0.77	-0.86

**p < .01 / ➀, ➁, ➂, ➃ (Observed variables for each latent variable)

The absolute values of skewness for the primary variables ranged from 0.28 to 1.03, while those of kurtosis ranged from 0.21 to 0.92, as indicated in Table 2. This confirms that these values satisfied the criteria for normality, with absolute values of skewness and kurtosis less than 2 and 7, respectively [38].

### Evaluation of the overall measurement model

The suitability of the reflective measurement model in this study was confirmed through convergent validity, internal consistency reliability, and discriminant validity. To establish convergent validity, the outer loading relevance ranged from .652 to .920, exceeding the standard value of .5 [39]. Additionally, the average variance extracted (AVE) ranged from .575 to .820, also surpassing the standard value of .5 [40]. These results satisfy the standard values, thereby ensuring convergent validity. To confirm internal consistency reliability, Cronbach’s α ranged from .755 to .890, exceeding the standard value of .7. Similarly, rho_A ranged from .773 to .891, and composite reliability from .843 to .932, both surpassing the standard value of .7. All these standards were met, thereby ensuring internal consistency reliability [41–43] (Table 3). Furthermore, as depicted in Table 4, the square root values of AVE were all greater than the correlation coefficient values between latent variables. This confirms that discriminant validity was achieved [40].

**Table 3:** Internal consistency reliability and convergent validity.

Latent variables		Outer loading	Cronbach’s α	rho_A	CR	AVE
**Parental respect for children’s decision-making**	**Decision on family issues**	.805	.822	.826	.882	.652
	**Decision on higher school**	.827				
	**Respect for career path**	.821				
	**Decision on study methods**	.774				
**Respect for human rights**	**At home**	.755	.755	.773	.843	.575
	**At school**	.830				
	**Within the broader society**	.784				
	**In digital spaces**	.652				
**Self-esteem**	**Own value**	.805	.793	.814	.876	.703
	**Own strengths**	.882				
	**Proud of myself**	.824				
**Depression**	**Loneliness**	.899	.890	.891	.932	.820
	**Anxious**	.896				
	**Sad or depressed**	.920				

CR = Composite reliability; AVE = Average variance extracted.

**Table 4:** Discriminant validity in the measurement model.

Latent variable	Parental respect for children’s decision-making	Respect for human rights	Self-esteem	Depression
**Parental respect for children’s decision-making**	.807			
**Respect for human rights**	.472	.758		
**Self-esteem**	.334	.391	.838	
**Depression**	-.206	-.339	-.370	.905

### Structural model results and hypothesis verification

In this study, the internal variance inflation factor of the structural model ranged from 1.391 to 2.885, falling below the standard value of 3 [44]. This indicates that multicollinearity was not an issue. The adjusted R^2^ values for self-esteem and depression were .221 and .257, respectively. The effect sizes (f^2^) of parental respect for children’s decision-making and respect for human rights on self-esteem were .038 and .070, respectively. Meanwhile, the effect sizes (f^2^) of parental attitudes towards decision-making, respect for human rights, and self-esteem on depression were .002, .046, and .060, respectively. The Q^2^ values for self-esteem and depression were .151 and .209, respectively, both exceeding 0. This confirms that the structural model demonstrated predictive suitability [45–47].

**Fig 2:**
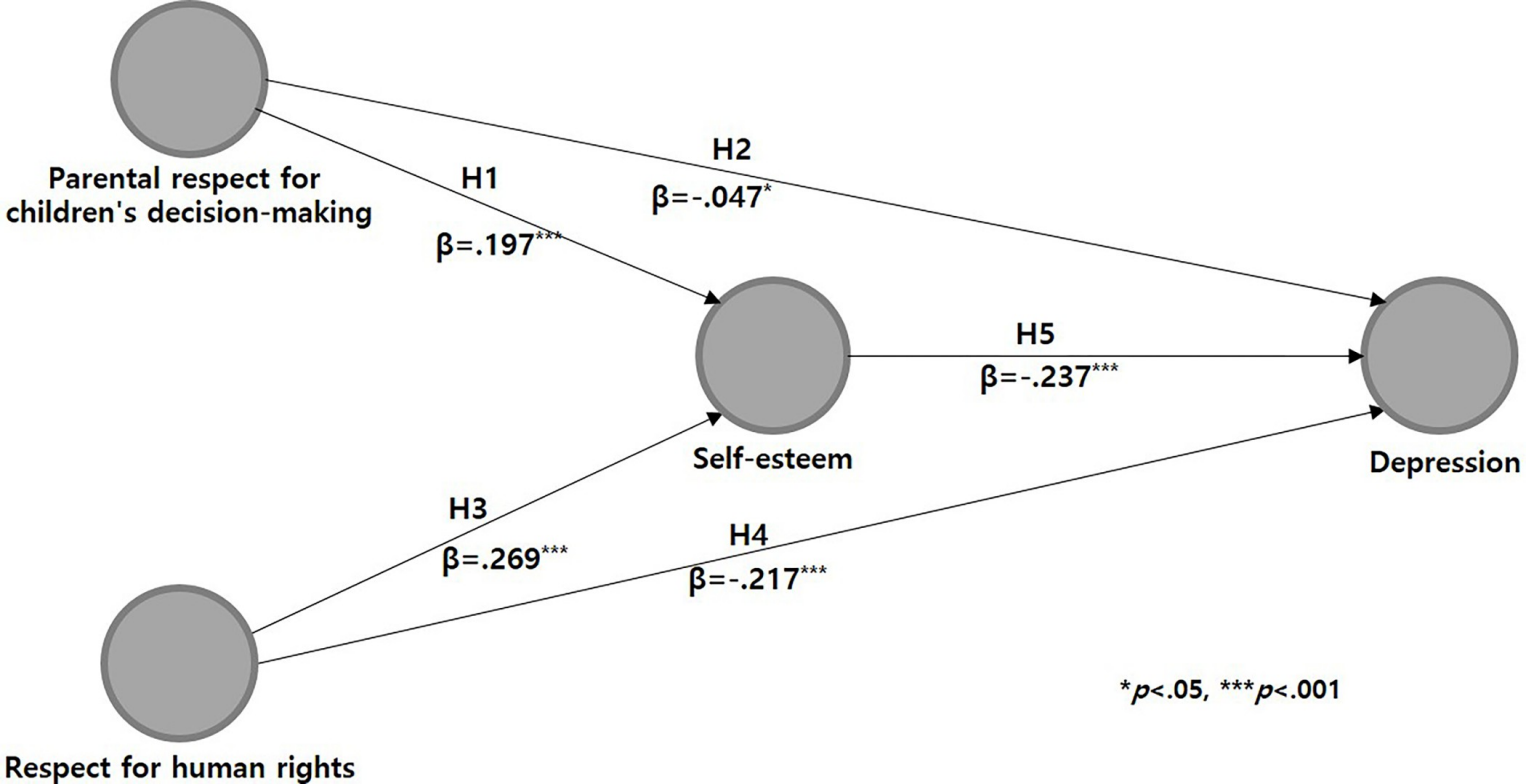
Results of hypothesis testing for the structural model.

The results of the hypothesis testing for the direct influence relationship of the structural model (Fig 2) revealed that parental respect for children’s decision-making significantly and positively influenced self-esteem (β = .197, p < .001). Conversely, these attitudes had a significant negative impact on depression (β = -.047, p = .029). Furthermore, the level of respect for human rights also showed a significant positive effect on self-esteem (β = .269, p < .001), and a significant negative effect on depression (β = -.217, p < .001). Additionally, self-esteem was found to have a significant negative effect on depression (β = -.237, p < .001).

In hypothesis testing on the relationships involving mediating influence in the structural model, parental respect for children’s decision-making (β = -.047, p < .001) and respect for human rights (β = -.064, p < .001) each showed a statistically significant negative mediating effect on depression, with self-esteem acting as a mediator. Gender, a control variable, exhibited a statistically significant effect on self-esteem (β = -.117, p < .001) and depression (β = .281, p < .001), but the perceived economic status only had a statistically significant effect on self-esteem (β = .160, p < .001) (Table 5).

**Table 5:** Research hypothesis testing.

Hypothesized path		Path coefficient	SD	t (*p*)
**Direct effects**				
**H1**	**Parental respect for children’s decision-making → Self-esteem**	.197	.021	9.48 (< .001)
**H2**	**Parental respect for children’s decision-making → Depression**	-.047	.021	2.20 (.029)
**H3**	**Respect for human rights → Self-esteem**	.269	.020	13.42 (< .001)
**H4**	**Respect for human rights → Depression**	-.217	.019	11.21 (< .001)
**H5**	**Self-esteem → Depression**	-.237	.020	11.96 (< .001)
**Control variables**	**Gender → Self-esteem**	-.117	.017	6.90 (< .001)
	**Gender → Depression**	.281	.015	18.50 (< .001)
	**Perceived economic status → Self-esteem**	.160	.018	8.71 (< .001)
	**Perceived economic status → Depression**	.000	.019	0.04 (.971)
**Indirect effects**				
**H6**	**Parental respect for children’s decision-making → Self-esteem → Depression**	-.047	.006	7.57 (< .001)
**H7**	**Respect for human rights → Self-esteem → Depression**	-.064	.007	9.09 (< .001)

## Discussion

This study utilized national data from the 2020 Survey on the Current Status of Korean Children’s and Youth’s Rights. The aim was to examine the structural relationship between parental respect for children’s decision-making, respect for human rights, and self-esteem, and their influence on depression in early adolescents. The research model was established on the basis of previous studies, using control variables such as gender and economic status. The following results were obtained from the analysis of the research model.

First, we found that parental respect for children’s decision-making significantly positively impacted the self-esteem of early adolescents (β = .197, p < .001). This finding aligns with several previous studies, which found that a respectful parental attitude towards decision-making had a significant positive effect on self-esteem of fourth- to sixth-grade elementary school students [33] and middle and high school students [12, 32]. This study underscores the importance of parents respecting their children’s decisions in fostering early adolescents’ self-esteem.

In addition, this study found that parental respect for children’s decision-making significantly reduced depression in early adolescents (β = -.047, p = .029). Our findings align with those of a previous study [12], which also found that parental respect for children’s decision-making significantly reduced academic and career stress in adolescents. This supports the idea that such parental attitudes are closely tied to adolescents’ psychological well-being. Our study also mirrors previous research that found positive, open communication with parents [48] and a high paternalistic attitude from parents [49, 50] can decrease depression. This further confirms that when parents adopt respectful and positive attitudes towards their children, it can help reduce depression in early adolescents. Recent trends in Korea show an improvement in parents’ child-rearing attitudes, including a growing respect for their adolescent children’s opinions in decision-making [3]. However, there is still a need to further emphasize parents’ respectful attitudes towards their children to enhance the psychological stability of early adolescents.

In particular, unlike Western cultures, where parents value their children’s autonomy and display individual and independent parent-child relationships, Korean parent-child relationships are characterized as being closer and stronger in solidarity due to a culture where relationships are valued, and so Korean parents have a greater influence on their children [51]. Therefore, in Korean parents, a greater emphasis should be placed on parental attitudes toward including their children in decision-making and respecting their children’s decision-making ability.

Second, we found that the level of respect for human rights significantly positively impacted the self-esteem of early adolescents (β = .269, p < .001). This finding aligns with several previous studies [32, 34, 35, 52], which reported a positive effect of respect for human rights on adolescents’ self-esteem. Furthermore, this study found that the level of respect for human rights, as perceived by early adolescents, significantly negatively affected depression (β = -.217, p < .001). This finding is consistent with the results of [34], who found that the level of respect for human rights perceived by youth had a significant negative impact on stress. Moreover, this study supports previous research indicating that human rights violations, such as parental abuse or neglect [16, 18, 19], social stigma [53, 54], and experiences of school violence and cyber victimization [17, 20] contribute to increased depression. In essence, it is crucial to emphasize the importance of respecting the human rights of youth in the home, school, and society. The results also suggest that, given the recent widespread use of personal PCs and mobile internet, youth need protection from human rights violations in cyberspace.

Third, this study found that self-esteem was the factor with the most significant negative impact on depression in early adolescents (β = -.237, p < .001). Our findings align with several previous studies [13, 24–27, 55, 56], which also demonstrated a significant negative relationship between adolescents’ self-esteem and depression. Given that self-esteem tends to decrease while depression tends to increase during adolescence [30], our results imply that a decline in self-esteem during this period can exacerbate the increase in depression. Furthermore, our findings are consistent with prior research showing that self-esteem significantly reduces academic and career stress in adolescents [12], and positively influences happiness [57] and life satisfaction [35]. Therefore, our study underscores the importance of initiatives to boost self-esteem for managing psychological issues such as depression in early adolescents.

Fourth, in this study, we examined the indirect effect of self-esteem on the relationships between parental respect for children’s decision-making and depression, and respect for human rights perceived and depression in early adolescents. We found that self-esteem significantly mediates both relationships. This suggests that a respectful parental attitude towards decision-making and human rights can enhance self-esteem, which in turn can reduce depression in early adolescents. While related research is scarce, our findings align with a similar study [33], which demonstrated a significant mediating effect of self-esteem on the relationship between parental respect for children’s decision-making and adolescents’ awareness of human rights. Our results also echo a previous study that found self-esteem significantly mediates the relationship between adolescents’ experiences of respect for human rights and life satisfaction [35]. Furthermore, our findings are consistent with research showing that self-esteem partially mediates the relationship between bullying victimization and depression among adolescents [58]. These results suggest that the more respect parents show for their children’s decision-making and the more respect for human rights adolescents perceive in their social environment, the higher their self-evaluation can be. This elevated self-esteem can contribute to a reduction in depression.

Gender, which was considered a control variable in this study, was found to significantly influence both self-esteem and depression. Economic status, on the other hand, only had a significant impact on self-esteem. These findings generally align with those of previous studies [12, 16, 17, 30, 32]. However, this study diverges from prior research in that it found economic status to be an insignificant factor in adolescent depression. This contrasts with previous studies that reported a significant relationship between economic status and both depression [3, 17] and stress [12] in adolescents. This discrepancy is interpreted as an indication that the perceived level of respect for human rights, the respectful attitude of their parents, and their self-esteem exert a stronger influence on adolescent depression than economic status alone.

This study is significant as it utilized national data to discern the structural relationship between variables associated with respect for human rights and self-esteem, both of which influence depression in early adolescents. Specifically, it is significant in identifying the comprehensive structural relationship between these variables by uncovering the indirect influence through self-esteem in the relationship between depression and variables related to perceived respect for human rights in early adolescents, a topic that has been seldom explored in previous studies. However, this study, which examined the structural relationship between variables using cross-sectional survey data from the first year, has limitations in establishing causal relationships. Therefore, future research should employ longitudinal data analysis to determine the causal relationship over time between adolescent depression, human rights-related variables, and self-esteem.

## Conclusion and suggestions

Based on the findings of this study, we make the following recommendations. Parental respect for children’s decision-making was identified as a factor that improved self-esteem and alleviated depression in early adolescents. Therefore, the importance of parents involving their adolescent children in decision-making and respecting their children’s opinions needs to be emphasized during parent education. The level of respect for human rights perceived by early adolescents was another factor that significantly influenced self-esteem and depression. This factor was found to have a greater impact on both self-esteem and depression than the parental attitude towards decision-making respect. In essence, the study indicates that the level of respect for human rights experienced in the home, school, and wider society plays a crucial role in the self-esteem and depression of early adolescents. Therefore, the importance of respecting the human rights of young people should be emphasized at a societal level. Instances of violations of youth human rights and guidelines for appropriate behavior respecting youth human rights should be made known.

There is also a pressing need to implement more robust measures to safeguard young people from cyber human rights violations. This issue has come to the fore as the use of personal computers and smartphones for internet access has surged among the youth. The 2020 Basic Analysis Report on the Human Rights Status of Children and Adolescents in Korea provides some insight into this matter. When asked about their perception of human rights respect, young people felt that their rights were less respected in Korea as a whole (16.3%) and in cyberspace (17.7%) compared to their homes (3.7%) or schools (4.4%). This suggests that the protection of youth human rights needs to be further emphasized not only in homes and schools, but also in society at large and in cyberspace [3]. Notably, awareness of human rights violations in cyberspace was the highest. Therefore, it is crucial to place greater emphasis on the development of relevant policies and education on cyber human rights protection to enhance societal awareness of cyber human rights violations.

In this study, self-esteem was not only identified as the most significant direct factor in adolescent depression, but also as a mediating factor that mitigates the negative effects of parental respect for children’s decision-making and respect for human rights on depression. Therefore, enhancing self-esteem is crucial for alleviating depression in adolescents. To achieve this, there is a need for a stronger emphasis on strategies that promote parental attitudes of respect for decision-making and human rights.

## References

[1] Thapar A, Collishaw S, Pine DS, Thapar AK. Depression in adolescence. Lancet. 2012;379(9820): 1056–1067. doi: 10.1016/S0140-6736(11)60871-4 22305766 PMC3488279

[2] Ministry of Education Kora Disease Control and Agency Prevention. 2022 Announcement of student health examination and youth health behavior survey results [Internet]. 2023 Apr 14 [cited 9 Nov 2023]. Available from: https://www.moe.go.kr/boardCnts/viewRenew.do?boardID=294&boardSeq=94695&lev=0&searchType=null&statusYN=W&page=1&s=moe&m=020402&opType=N2023.

[3] Kim YJ, Hwang SY, Choi HG. 2020 Implementation Study of the International Convention on the Rights of Children and Adolescents—The Human Rights Situation of Children and Adolescents in Korea: Basic Analysis Report. Sejong: Korea Youth Policy Institute; 2020.

[4] Piko BF. Adolescent life satisfaction: Association with psychological, school-related, religious and socially supportive factors. Children (Basel). 2023;10(7): 1176. doi: 10.3390/children10071176 37508673 PMC10378027

[5] Son WJ, Bae SM. The relationship between human rights, negative affect, bullying victimization, and life satisfaction among Korean adolescents: A national sample study. Child Youth Serv Rev. 2022;139: 106568. doi: 10.1016/j.childyouth.2022.106568

[6] Grossberg A, Rice T. Depression and Suicidal Behavior in Adolescents. Med Clin North Am. 2023;107(1): 169–182. doi: 10.1016/j.mcna.2022.04.005 36402497

[7] Patton GC, Viner R. Pubertal transitions in health. Lancet. 2007;369(9567): 1130–1139. doi: 10.1016/S0140-6736(07)60366-3 17398312

[8] Gillham JE, Brunwasser SM, Freres DR. Preventing depression in early adolescence: The penn resiliency program. Handbook of Depression in Children and Adolescents. NY: Guilford Press; 2008. pp. 309–322.

[9] Kieling C, Adewuya A, Fisher HL, Karmacharya R, Kohrt BA, Swartz JR, et al. Identifying depression early in adolescence. Lancet Child Adolesc Health. 2019;3(4): 211–213. doi: 10.1016/S2352-4642(19)30059-8 Erratum in: Lancet Child Adolesc Health. 2019;3(6): e4. 30878111 PMC6849697

[10] Kim SA. The effects of parent abuse, negative peer relations, and cell phone dependency on middle-school adolescent depression. Forum for Youth Culture. 2015;43: 31–56.

[11] Sung JM. The longitudinal factors on depression in Korean adolescents. Korean Journal of Youth Welfare. 2016;18(4): 93–111. doi: 10.19034/KAYW.2016.18.4.05

[12] Lee EB, Kim JW. The relationship between parenting respecting children’s decision-making and adolescent’s attitude towards life: Focusing on the mediating effects of self-esteem and academic and career stress. Korean Journal of Youth Studies. 2022;29(4): 137–164. doi: 10.21509/KJYS.2022.04.29.4.137

[13] Acun-Kapikiran N, Körükçü Ö, Kapikiran S. The relation of parental attitudes to life satisfaction and depression in early adolescents: The mediating role of self-esteem. Educ. 2014;14(4): 1246–1252.

[14] United Nations General Assembly. The Universal Declaration of Human Rights (UDHR). New York: United Nations General Assembly; 1948.

[15] National Center for the Rights of the Child. UN Convention on the Rights of the Child (CRC). [Internet]. 2023 Apr 14 [cited 9 Nov 2023]. Available from: http://www.korea1391.go.kr/new/page/agreement.php.

[16] Kim SA, Bae SM. Impacts of perceived stress, neglect, victim and respect for human rights on depression of adolescents. Child Psychiatry Hum Dev. 2023. doi: 10.1007/s10578-023-01491-3 36645536

[17] Oh HK. The Effect of adolescents’ experience of school violence and cyberbullying on depression: Focused on mediating effect of respect of human rights in school and home. Korean Journal of Convergence Science. 2022;11(4):169–183. doi: 10.24826/KSCS.11.4.11

[18] Infurna MR, Reichl C, Parzer P, Schimmenti A, Bifulco A, Kaess M. Associations between depression and specific childhood experiences of abuse and neglect: A meta-analysis. J Affect Disord. 2016;190: 47–55. doi: 10.1016/j.jad.2015.09.006 26480211

[19] Zhao J, Sun X, Wang Q. Emotional neglect and depressive symptoms of left-behind adolescents: The role of friendship quality and gender. J Affect Disord. 2021;295: 377–383. doi: 10.1016/j.jad.2021.08.073 34492430

[20] Tran HGN, Thai TT, Dang NTT, Vo DK, Duong MHT. Cyber-victimization and its effect on depression in adolescents: A systematic review and meta-analysis. TVA. 2023;24(2): 1124–1139. doi: 10.1177/15248380211050597 34689637

[21] Zeiders KH, Umana-Taylor AJ, Derlan CL. Trajectories of depressive symptoms and self-esteem in Latino youths: Examining the role of gender and perceived discrimination. Dev Psychol. 2013;49(5): 951. doi: 10.1037/a0028866 22686175

[22] Lavner JA, Ong ML, Carter SE, Hart AR, Beach SRH. Racial discrimination predicts depressive symptoms throughout adolescence among Black youth. Dev Psychol. 2023;59(1): 7–14. doi: 10.1037/dev0001456 36066872 PMC9822848

[23] Rosenberg M. Society and the adolescent self-image. Princeton, NJ: Princeton University Press; 1965.

[24] Orth U, Robins RW. Understanding the link between low self-esteem and depression. Curr Dir Psychol Sci. 2013;22(6): 455–460. doi: 10.1177/0963721413492763

[25] Sowislo JF, Orth U. Does low self-esteem predict depression and anxiety? A meta-analysis of longitudinal studies. Psychol Bull. 2013;139(1): 213–240. doi: 10.1037/a0028931 22730921

[26] Orth U, Robins RW, Widaman KF, Conger RD. Is low self-esteem a risk factor for depression? Findings from a longitudinal study of Mexican-origin youth. Dev Psychol. 2014;50(2): 622–633. doi: 10.1037/a0033817 23895172 PMC3815504

[27] Zhou J, Li X, Tian L, Huebner ES. Longitudinal association between low self-esteem and depression in early adolescents: The role of rejection sensitivity and loneliness. Psychol Psychother. 2020;93(1): 54–71. doi: 10.1111/papt.12207 30488549

[28] Steiger AE, Allemand M, Robins RW, Fend HA. Low and decreasing self-esteem during adolescence predict adult depression two decades later. J Pers Soc Psychol. 2014;106(2): 325–338. doi: 10.1037/a0035133 24467425

[29] Masselink M, Van Roekel E, Oldehinkel AJ. Self-esteem in early adolescence as predictor of depressive symptoms in late adolescence and early adulthood: The mediating role of motivational and social factors. J Youth Adolesc. 2018;47(5): 932–946. doi: 10.1007/s10964-017-0727-z 28785953 PMC5878202

[30] Robins RW, Kali HT. Self-esteem development across the lifespan. Curr Dir Psychol Sci. 2005;14(3): 158–162. doi: 10.1111/j.0963-7214.2005.00353.x

[31] Singh S. Parenting style in relation to children’s mental health and self-esteem: A review of literature. IJHW. 2017;8(12): 1522–1527.

[32] Lee EB, Noh JE, Lim SH, Lee SW. How do parenting attitudes respecting children’s decision-making rights affect children’s human rights awareness?—Focusing on the roles of experience of children’s rights being respected in home and self-esteem. Korean Journal of Youth Studies. 2022;29(11): 377–406. doi: 10.21509/KJYS.2022.11.29.11.377

[33] Jeong YM. Respect your child’s decisions Effect of parental attitude on human rights awareness: Mediating Effect of Self-Esteem. The Journal of the Convergence on Culture Technology. 2022;8(2): 77–82. doi: 10.17703/JCCT.2022.8.2.77

[34] Park MH, Choe WS. Influence of human rights respect and stress of adolescents on their self-concept. Studies on Life and Culture. 2017;44: 109–133.

[35] Park SJ, Park KA. (2020). The effect of youth s respect for human rights on life satisfaction: Mediating effect of self-esteem. JLCCI. 2020;20(22): 349–365. doi: 10.22251/jlcci.2020.20.22.349

[36] Palermiti AL, Servidio R, Bartolo MG, Costabile A. Cyberbullying and self-esteem: An Italian study. Comput Hum Behav. 2017;69: 136–141. doi: 10.1016/j.chb.2016.12.026

[37] Patchin JW, Hinduja S. Cyberbullying and self-esteem. J Sch Health. 2010;80(12): 614–621. doi: 10.1111/j.1746-1561.2010.00548.x 21087257

[38] Curran PJ, West SG, Finch JF. The robustness of test statistics to nonnormality and specification error in confirmatory factor analysis. Psychol Methods. 1996;1(1): 16–29.

[39] Chin WW. The partial least squares approach to structural equation modeling. Modern Methods for Business Research. 1998;295: 295–336.

[40] Fornell C, Larcker DF. Evaluating structural equation models with unobservable variables and measurement error. J Mark Res. 1981;18(1): 39–50.

[41] Cronbach LJ. Coefficient alpha and the internal structure of tests. Psychometrika. 1951;16: 297–334.

[42] Dijkstra TK, Henseler J. Consistent partial least squares path modeling. MIS Quarterly 2015;39(2): 297–316.

[43] Werts CE, Linn RL, Jöreskog KG. Intraclass reliability estimates: Testing structural assumptions. Educ Psychol Meas. 1974;34(1): 25–33.

[44] Hair JF, Hult GTM, Ringle CM, Sarstedt M. A primer on partial least squares structural equation modeling (PLS-SEM). 3 Ed. Thousand Oaks: Sage; 2022.

[45] Fornell C, Cha J. Partial least squares. In advanced methods of marketing research: Bagozzi R, Ed. Cambridge: Blackwell Business; 1994.

[46] Stone M. Cross‐validatory choice and assessment of statistical predictions. J R Stat Soc B: Stat Methodol. 1974;36(2): 111–133.

[47] Geisser S. A predictive approach to the random effect model. Biometrika. 1974;61: 101–107.

[48] Yeoum SG, Lee JH. The influences on self-esteem and depression of parent-adolescent communication for the adolescent. Korean Public Health Research. 2014;40(3): 1–11.

[49] DeHart T, Pelham BW, Tennen H. What lies beneath: Parenting style and implicit self-esteem. J Exp Soc Psychol. 2006;42(1): 1–17. doi: 10.1016/j.jesp.2004.12.005

[50] Park SS, An GYR. The effect of parenting attitude, self-esteem, and social support on adolescents’ depression. Korean Journal of Youth Studies. 2023;30(6): 339–366. doi: 10.21509/KJYS.2023.06.30.6.339

[51] Choi IJ. Cultural psychological implication of the Korea parent-child relationship. Korea Journal of Counseling. 2006;7(3): 761–773.

[52] Cheon JW. A structural analysis on the youth consciousness of human rights: Respect for human rights, human rights education, discriminating experiences, and self-esteem. Journal of Future Oriented Youth Society. 2015;12(4): 1–23.

[53] Park DJ, Kim NY. Mediating effects of self-esteem on the effects of social stigma on depression of out-of-school adolescents. The Journal of Youth Activity. 2019;5(3): 71–87.

[54] Kwon T. Social stigma, ego-resilience, and depressive symptoms in adolescent school dropouts. J Adolesc. 2020;85: 153–163. doi: 10.1016/j.adolescence.2020.11.005 33246287

[55] Berber Çelik Ç, Odacı H. Does child abuse have an impact on self-esteem, depression, anxiety and stress conditions of individuals? Int J Soc Psychiatry. 2020;66(2): 171–178. doi: 10.1177/0020764019894618 31856622

[56] MacPhee AR, Andrews JJ. Risk factors for depression in early adolescence. Adolescence. 2006;41(163): 435–466. 17225661

[57] Dogan T, Totan T, Sapmaz F. The role of self-esteem, psychological well-being, emotional self-efficacy, and affect balance on happiness: A path model. Eur Sci J. 2013;9(20): 31–42.

[58] Zhong M, Huang X, Huebner ES, Tian L. Association between bullying victimization and depressive symptoms in children: The mediating role of self-esteem. J Affect Disord. 2021;294: 322–328. doi: 10.1016/j.jad.2021.07.016 34311332

